# *CpARF6* Controls Lobed Leaf Formation in Zucchini

**DOI:** 10.3390/ijms262010042

**Published:** 2025-10-15

**Authors:** Shufang Jiang, Lu Shi, Shuliang Fei, Mengyi Zhang, Yimei Zhou, Zhongyuan Hu, Jinghua Yang, Mingfang Zhang, Xiaolong Lyu

**Affiliations:** 1 Laboratory of Germplasm Innovation and Molecular Breeding, Institute of Vegetable Science, Zhejiang University, Hangzhou 310058, China; a982805848@163.com (S.J.);; 2 Hainan Institute, Zhejiang University, Sanya 572025, China; 3 Key Laboratory of Vegetable Germplasm Innovation and Quality, Breeding in the Province, Hangzhou 310058, China

**Keywords:** zucchini, lobed leaves, gene mapping, BSA-seq, ARF

## Abstract

Lobed leaves play a critical role in enhancing the productivity of sprawling crops like zucchini by improving light capture and boosting photosynthesis. However, the genetic basis in zucchini remains largely unknown. Here, we developed an F_2_ population from a cross between the entire-leaf cultivar ‘LR’ and the deeply lobed cultivar ‘Xi’. Genetic analysis showed that the non-lobed trait is dominant, with the F_2_ segregation ratios (~9 entire:6 shallowly lobed:1 deeply lobed) indicating digenic inheritance. Using bulked segregant analysis sequencing (BSA-seq) and kompetitive allele-specific PCR (KASP) marker analysis, we identified a major effect locus at a 79.8 kb interval on chromosome 10. Within in this interval, gene expression profiling and annotation indicated *CpARF6*, encoding an auxin response factor, to be the prime candidate gene. Sequencing analysis revealed five nonsynonymous mutations in this gene, including a critical serine-to-leucine substitution at position 335 within the auxin response domain, which is likely a loss function mutation. Our findings establish *CpARF6* as a critical regulator of lobed leaf formation in zucchini, providing valuable insights for both leaf development studies and zucchini breeding.

## 1. Introduction

Leaves serve as the primary organs for photosynthesis and gas exchange in plants, with their morphological characteristics directly influencing environmental adaptability [1]. Through the long-term evolutionary process, plants have developed diverse leaf margin morphologies, including entire, shallowly, and deeply lobed forms [2]. These morphological variations contribute to enhanced plant adaptation by improving light capture efficiency, increasing heat dissipation surface area, and optimizing water use efficiency [3,4], conferring distinct advantages to lobed leaf plants under drought and high-temperature stress [5]. In agricultural production, leaf morphology directly affects crop yield by regulating the canopy architecture and light utilization efficiency [6]. Therefore, unraveling the regulatory mechanisms underlying leaf lobe formation holds significant theoretical and applied value.

The formation of leaf lobes is initiated by the establishment of adaxial–abaxial polarity. This process relies on spatial antagonism between adaxial factors, such as class III Homeodomain-Leucine Zipper (HD-ZIP) transcription factors, including *PHABULOSA* (*PHB*), *PHAVOLUTA* (*PHV*), and *REVOLUTA* (*REV)*, and abaxial factors, such as GARP family transcription factors, including *KANADI 1*, *2*, *3*, and *4* (*KAN1–4*) [7,8]. In the shoot apical meristem, *HD-ZIP III* genes are expressed in the central (adaxial) domain, whereas *KAN* genes exhibit expression in the peripheral (abaxial) region. Enhanced abaxial identity promotes abaxial cell proliferation and expansion, causing leaf curling or lobe formation. In contrast, a dominant adaxial identity inhibits lobing and promotes smooth margins. This polarity system directs auxin distribution via PIN-FORMED (PIN)-mediated transport, establishing auxin maxima at the leaf margin that determine the lobe initiation sites [9,10].

Auxin maxima activate auxin response factor (ARF) by degrading auxin/indole-3-acetic acid (Aux/IAA) repressors [11]. The activated ARFs bind to auxin response elements (AuxREs) in the promoters of downstream genes, directly regulating cell proliferation, differentiation, and expansion [12,13]. Concurrently, CUP-SHAPED COTYLEDON (CUC) stabilizes the polar localization of PIN proteins to maintain the auxin maxima, forming a self-sustaining PIN–auxin–CUC feedback loop that ensures robust patterning of serrated margins [14]. The precise spatial expression of *CUC* in marginal sinus regions is tightly controlled through transcriptional and post-transcriptional repression. At the transcriptional level, TEOSINTE BRANCHED1/CYCLOIDEA/PCF (TCP) factors directly suppress *CUC* expression and simultaneously antagonize auxin signaling, which restrains lobe formation [15,16]. miR164 acts post-transcriptionally to reduce CUC accumulation, thereby preventing excessive serration [9,17]. In contrast, KNOTTED1-LIKE HOMEOBOX (KNOX) transcription factors interact with the PIN–auxin–CUC module by modulating hormone homeostasis to delay differentiation and promote leaflet initiation [18,19]. Together, these interactions form a precise regulatory network that determines the plasticity and complexity of leaf margin morphology.

The complex network of leaf lobe formation stems from the coordinated action of multiple key genes. In recent years, researchers have identified critical regulators of leaf morphogenesis across various crop species. Notably, studies on leaf lobe development are particularly advanced in cruciferous plants. For instance, in the model organism *Arabidopsis*, WOX1 regulates the expression of *BZR1* and *CUC3* to control serrated leaf margin formation [14]. Similarly, in rapeseed (*Brassica napus*), a major QTL on chromosome A10 harbors *BnaA10g26320D/26330D*, which promotes lobe formation via the auxin pathway [20], whereas the WIP2–STM module fine-tunes leaf margin patterning by integrating cytokinin [21]. In cabbage (*Brassica rapa*), *BrACP5* and *BrLMI1* influence leaf shape variation through altered auxin response activity [1,22]. Furthermore, in ornamental kale (*Brassica oleracea*), *BoFL* directs lobe formation through the adaxial–abaxial polarity pathway [23]. Beyond cruciferous species, key leaf shape regulators have also been identified in numerous other plant families. Notable examples include *L* and *GhRl4* in upland cotton (*Gossypium hirsutum*) [19,24], *Vradi03g04470* in *Vigna radiata* [25], *LtuHB6* in tulip tree (*Liriodendron tulipifera*) [26], as well as *SALAD* and *SL1* in strawberry (*Fragaria vesca*) [27]. Research on cucurbit crops has also made significant progress in the genetic mapping of key regulators underlying leaf morphological diversity. In watermelon (*Citrullus lanatus*), several loci have been identified, including *ClLMI1* [28] for lobe formation, *ClLL1* (Yan et al., 2025) for leaf development, and *ClLs* [6] for leaf shape and organogenesis. Similarly, in melon (*Cucumis melo*), the *pll* locus [29] has been genetically mapped for its role in palmate lobe formation. In cucumber (*Cucumis sativus*), multiple genetic determinants have been localized, such as *scl1* [30] for heart-shaped leaves, *Csa1M537400* [31] for round leaves, and *CsPHB* [32] for leaf curling, illustrating the coordinated genetic control of leaf morphology in the species. Moreover, in zucchini (*Cucurbita pepo*), *Cpdll* was mapped to a 21 kb region on chromosome 10 and is recognized as an incompletely dominant gene for leaf lobation [7]. Although our study initially identified overlapping genomic intervals, fine mapping ultimately revealed distinct candidate genes.

In zucchini, the deeply lobed leaf trait improves stress resilience and light utilization. This morphology enhances canopy ventilation and light penetration, supporting high-density planting for increased yield potential [7]. Therefore, in this study, we constructed an F_2_ population using the smooth-leaf LR and deeply lobed Xi zucchini varieties as parents. Through BSA-seq combined with fine mapping, we identified a key candidate gene, *CpARF6*, controlling leaf lobing in zucchini. A serine-to-leucine substitution within its auxin response domain coincides with loss of function. This discovery provides new insights into the molecular mechanism of zucchini leaf lobing, while also offering a valuable target gene for research on zucchini leaf margin development.

## 2. Results

### 2.1. Lobed Leaf Trait in Zucchini Is Controlled by Two Recessive Genes

**Figure 1:**
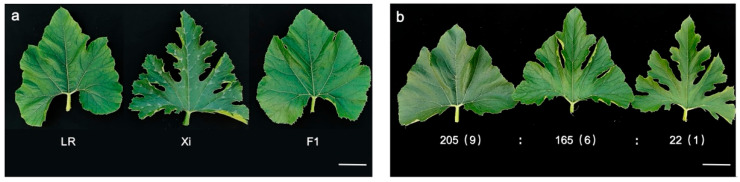
Phenotypic features of the mature leaves of the selected parents, their F_1_ hybrid and F_2_ population. (**a**) Leaf features of the entire parent (LR), deeply parent (Xi) and F_1_. (**b**) Phenotypic segregation of F_2_. F_2_ leaves exhibit three phenotypes: entire leaf (left), shallowly lobed leaf (middle), and deeply lobed leaf (right). Scale bars, 5 cm.

To investigate the inheritance pattern of the lobed leaf trait in zucchini, the entire leaf accession and lobed leaf accession were selected as parents to construct a population. The parental lines exhibited distinct leaf morphologies: LR showed entire (non-lobed) leaves, while Xi displayed deeply lobed leaves in field conditions. The F_1_ generation uniformly presented entire leaves (Figure 1a), phenotypically identical to the LR parent, suggesting that the lobed leaf trait is recessively inherited. We performed a phenotypic characterization of 392 F_2_ progeny through visual assessment in field conditions. The leaf margin phenotypes showed clear qualitative variation in lobation depth, allowing unambiguous classification. Comparative analysis revealed three distinct classes: 205 with entire leaves, 165 with shallowly lobed leaves, and 22 deeply lobed leaves, approximating a 9:6:1 ratio. The leaf morphology became distinguishable at the third true leaf stage, with clear differentiation by the fifth leaf stage (Figure 1b). Chi-square analysis confirmed that the observed segregation ratio (9 entire:6 shallowly lobed:1 deeply lobed) matched the expected ratio (χ^2^ = 3.549, χ^2^_0.05_ = 5.991; Figure 1b, Table 1). These results suggest that the leaf lobation trait is controlled by two recessive genes.

### 2.2. Gene Mapping of the Candidate Locus for the Lobed Leaf Trait

**Figure 2:**
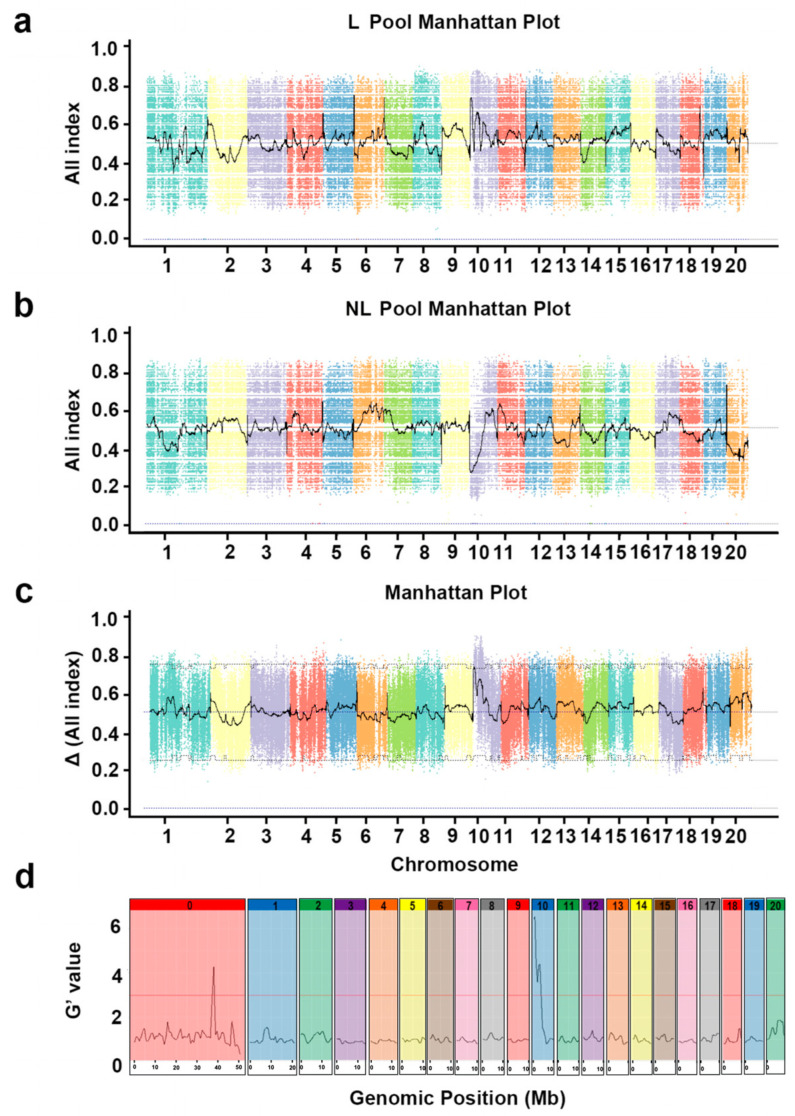
Gene mapping for the lobed leaf trait. (**a**) SNP-index plot of the lobed leaf pool (L pool), a DNA pool constructed by mixing individuals with the lobed leaf phenotype. The purple dashed line indicates the All Index of 0. (**b**) SNP-index plot of the non-lobed leaf pool (NL pool), a DNA pool constructed by mixing individuals with the non-lobed leaf phenotype. The purple dashed line indicates the All Index of 0. (**c**) Δ(SNP-index) for association analysis. The *x*-axis and *y*-axis represent the 20 chromosomes and SNP-index of zucchini, respectively. The solid black line represents the fitted SNP-index or Δ(SNP-index). The black dashed line represents the threshold lines of the 95% confidence interval. The purple dashed line indicates the Δ (All Index) of 0. (**d**) Major quantitative trait loci for the lobed leaf trait identified by QTLseqr. Different numbers represent distinct chromosomal intervals. The horizontal red line represents the threshold (10^−5^).

To identify candidate loci controlling the lobed leaf trait in zucchini, we performed BSA-seq using the parental lines (LR and Xi) and extreme phenotype pools from the F_2_ population (non-lobed pool and lobed leaf pool), each sequenced to approximately 10× genome coverage. Whole-genome resequencing was conducted to facilitate preliminary quantitative trait locus (QTL) mapping. Sequencing of the lobed leaf–entire leaf bulks was performed using the Illumina HiSeq4000 platform, generating 24.038 G of clean data. The majority of the obtained data were of high quality, with Q20 ≥ 96.38%, Q30 ≥ 90.84%, and the G/C ratios ranging from 38.55% to 40.02% (Table S3). After removing duplicates, the single nucleotide polymorphism (SNP) indices of the L pool (lobed leaf pool) and NL pool (non-lobed leaf pool) were calculated, and frequency distribution maps of the progeny SNPs across each chromosome were generated (Figure 2a,b). Subsequently, Δ(SNP-index) was obtained by subtracting the SNP-index of the N pool from that of the NL pool, and peak regions exceeding the threshold were selected as candidate regions for QTLs associated with the target trait (Figure 2c). At the 95% confidence level, one major candidate region was identified on chromosome 10. However, based on the trait segregation ratio of the F_2_ population, the lobed leaf trait should be regulated by two pairs of genes. Therefore, we used QTL Sequencing R package (QTLseqr) to analyze the data, including the reads mapped to the scaffolds (shown as chromosome 0), and calculated G’ values mapped to the genomic position. At the 99% significance level, two genomic regions, Cp4.1LG10: 204700-3790420 and Cp4.1LG00: 37128116-38176795, showed G’ values exceeding the threshold (Figure 2d). However, since the genes on the scaffolds (chromosome 0) have not been accurately positioned in the zucchini genome, fine mapping could not be performed. Therefore, we next focused on fine mapping the target genes within the candidate region on chromosome 10. The peak regions from the two mapping results overlapped in the 3.42 Mb region (Cp4.1LG Chr10: 204700-3790420; 95% significance level), further supporting that the candidate gene is likely located in this region (Table S4).

### 2.3. Fine Mapping of the Candidate Locus for the Lobed Leaf Trait

**Figure 3:**
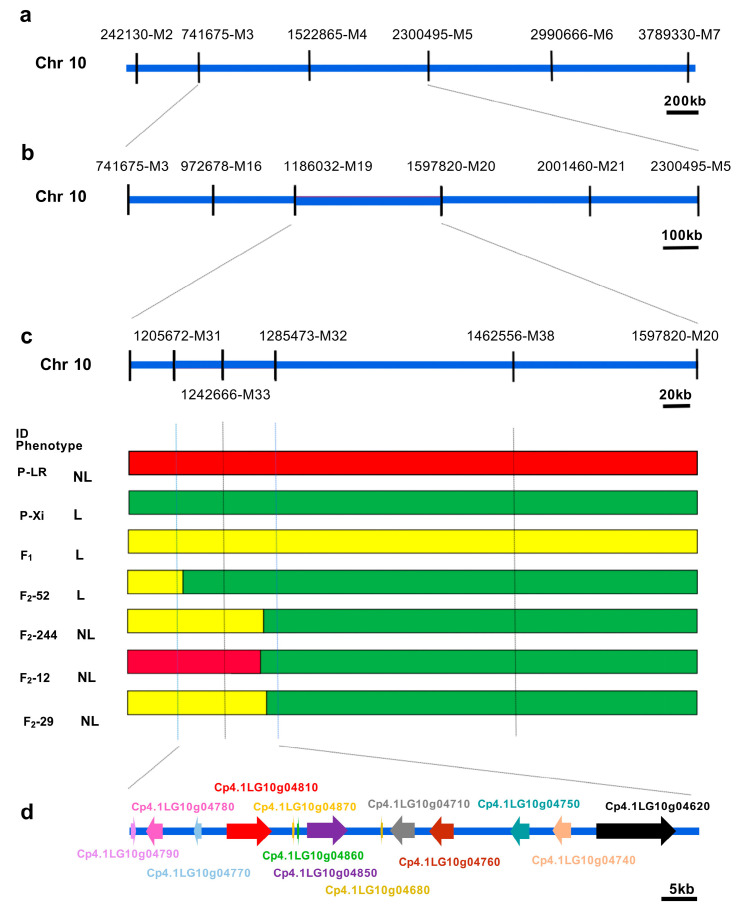
Fine mapping of the candidate gene for the lobed leaf trait. (**a**) Preliminary localization of the target region using 392 F_2_ individuals. Candidate genes were identified between markers 741675-M3 and 2300495-M5. (**b**) Fine mapping of the candidate genes. The candidate gene is located in the 411.8 kb region between the 1186032-M19 and 1597820 markers. (**c**) Individuals with chromosome segment substitution on the target region. Red boxes indicate the genotype of LR, green boxes indicate the genotype of Xi, and the yellow boxes indicate the genotype of the heterozygous region. (**d**) Candidate genes in the target region. The arrows indicate genes, and the dark blue lines represent the intergenic regions and promoter regions.

To further refine the candidate genes controlling the lobed leaf trait, we designed KASP markers based on the BSA-seq results and constructed a genetic map of the target region to narrow down the candidate interval (Figure 3a–c). SNP markers were extracted from the variant call format (VCF) files generated by genetic map analysis. Initially, 21 KASP markers were designed to genotype F_2_ individuals, after which the candidate interval was narrowed down to the region between 1186032-M19 and 1597820-M20, and 28 recombinants were screened in this region. Subsequently, we developed additional KASP markers and successfully delineated a 79.8 kb target region located between markers 1205672M31 and 1285473M32 (Figure 3c). Based on the zucchini genome, 13 candidate genes were annotated within the refined region (Figure 3d, Table S5). QTL-seq analysis of the parental lines revealed that all 13 candidate genes contained nonsynonymous SNPs and insertions and deletions (InDels).

### 2.4. Expression Profiles of Genes in the Candidate Region

**Figure 4:**
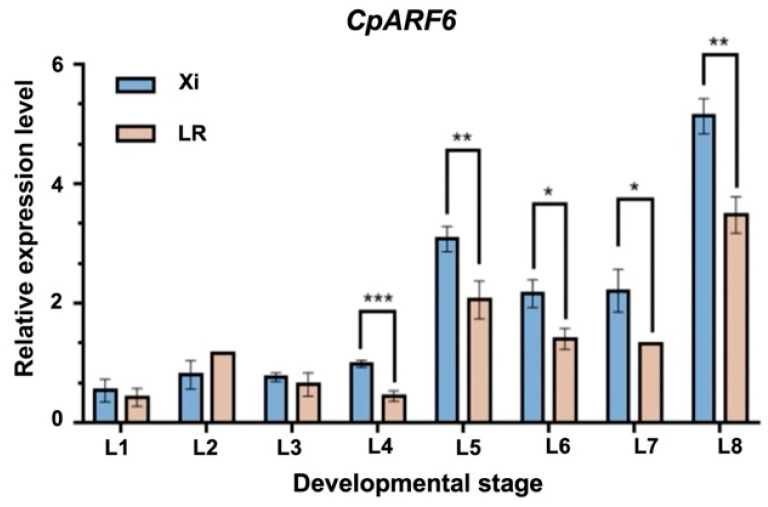
Expression analysis of the candidate gene *CpARF6*. L1–L8 represents the first true leaf development stage; ‘Xi’ and ‘LR’ represent two parents; ‘*’ indicates *p* < 0.05, ‘**’ indicates *p* < 0.01, ‘***’ indicates *p* < 0.001. Statistical analysis using one-way ANOVA. Three biological replicates were set up in the experiment.

To identify the key candidate genes regulating the lobed leaf trait, we conducted spatiotemporal expression analyses of 13 genes within the candidate region. We collected the first eight true leaves from both parental lines for qPCR analysis. The results showed that the expression levels of *Cp4.1LG10g04790*, *Cp4.1LG10g04770*, *Cp4.1LG10g04810*, *Cp4.1LG10g04870*, *Cp4.1LG10g04860*, *Cp4.1LG10g04850*, *Cp4.1LG10g04680*, *Cp4.1LG10g04710*, *Cp4.1LG10g04750*, *Cp4.1LG10g04740*, and *Cp4.1LG10g04620* in LR were significantly higher than those in Xi across multiple developmental stages (Figure S1). Overall, these genes exhibited an S-shaped fluctuation or a declining trend after reaching a peak. Additionally, the expression of *Cp4.1LG10g04780* was higher in Xi during the first five leaves, but by the seventh leaf, its expression level in LR became significantly higher than that in Xi (Figure S1). The leaf morphology of the two parental lines showed no significant differences in the first two true leaves. Phenotypic divergence began to emerge from the third true leaf onward, and the differences became highly pronounced after the fifth leaf. These findings indicate that the expression patterns of the above-mentioned genes are not correlated with the process of leaf lobe development. Notably, *Cp4.1LG10g04760* showed no consistent trend in expression fluctuation between the two parents at early stages. However, from the third to the eighth true leaf, its expression generally increased and remained significantly higher in Xi than in LR (Figure 4). The expression dynamics of this gene were highly consistent with the developmental progression of leaf lobing in the parents. Annotation analysis indicated that *Cp4.1LG10g04760* encodes ARF6, which is involved in auxin signal transduction and leaf development regulation. While this evidence is correlative and derived from parental lines, we propose that *CpARF6* is a key candidate gene regulating the formation of leaf lobing.

### 2.5. Non-Synonymous Variation of CpARF6 Confers the Lobed Leaf Trait

**Figure 5:**
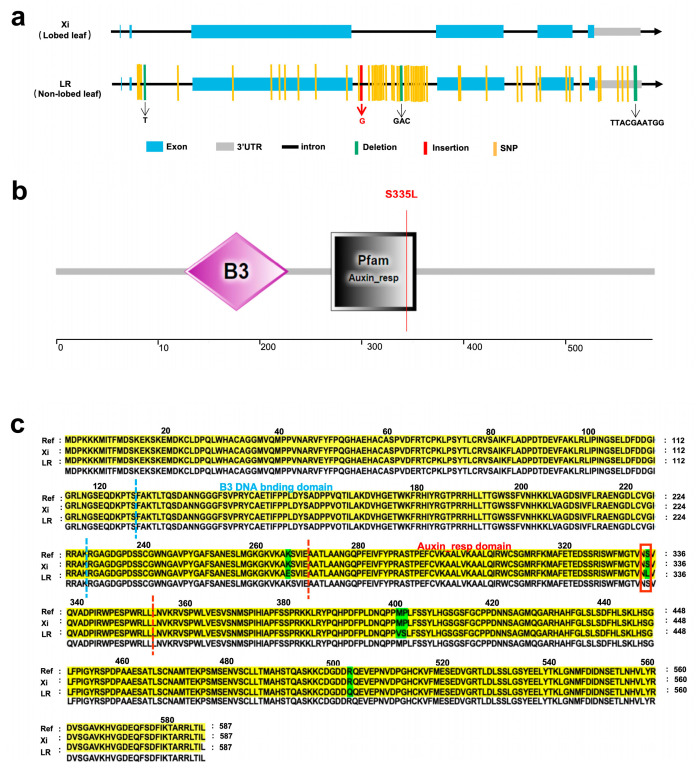
Sequence variations of the candidate gene *CpARF6*. (**a**) Gene structure and sequence variations of the candidate gene *CpARF6* between parents LR and Xi. Black horizontal lines represent introns, green vertical lines represent deletions, red vertical lines represent insertions, and yellow vertical lines represent SNPs. (**b**) Protein structure of ARF6. Purple and black boxes indicate the domain region. (**c**) Protein sequence alignment of *CpARF6* between parents LR and Xi. The green parts represent five nonsynonymous mutations. The red box highlights the S335L substitution.

In order to further investigate the genetic basis of the lobed leaf trait, we cloned the *CpARF6* gene and analyzed the sequence variations between the two parents. The *CpARF6* gene consists of six exons (Figure 5a) and encodes a protein composed of 587 amino acids. Sequence analysis between the LR and Xi parental lines showed multiple SNPs and InDels in both intronic and exonic regions of the candidate gene *CpARF6*. The genomic DNA sequences of *CpARF6* in LR and Xi were 3451 bp and 3464 bp, respectively (Figure S2). There were 57 SNPs and four InDels in the intron, exon and 3′UTR regions. Within the coding region, we detected 12 SNPs, including five nonsynonymous mutations: K265E (lysine to glutamic acid), S335L (serine to leucine), M400V (methionine to valine), P401S (proline to serine), and R502Q (arginine to glutamine) (Figure 5c). The putative protein contains an auxin response factor domain and a B3 DNA-binding domain (DBD) (Figure 5b). Notably, the S335L substitution in the LR parent occurred within the auxin-responsive domain of the protein, which could potentially cause structural alterations and functional abnormalities. To investigate the evolutionary and functional conservation of this gene, we extracted 22 protein sequences homologous to the candidate gene *CpARF6* from *Arabidopsis thaliana* and other plant species. We then performed multiple sequence alignment and constructed a phylogenetic tree. These results revealed that this homologous gene is widely present in seven cucurbit crops, including *Cucurbita moschata*, *Cucurbita maxima*, *Cucurbita argyrosperma*, *Cucumis melo*, *Cucumis sativus*, *Citrullus lanatus*, and *Benincasa hispida*, as well as in *Arabidopsis* (Figure S3). Further sequence alignment showed that all these homologous proteins, including the *Arabidopsis* orthologue, exhibit a high degree of sequence conservation (Figure S4), strongly suggesting that their function may be highly conserved across species.

## 3. Discussion

Leaf morphology is a crucial phenotypic trait for environmental adaptation in plants, with its variation profoundly influencing physiological functions, stress resistance, and breeding applications [27]. As a key component of leaf shape diversity, lobed leaves’ lobation not only enhances the cooling capacity under high temperatures and improves the drought tolerance [33] but also serves as an effective morphological marker in hybrid breeding [20]. In cucurbit crops, the genetic regulation of leaf lobing exhibits significant complexity and species specificity. For example, in *Cucurbita maxima*, lobed leaves are controlled by a recessive gene *lo-1* [34], while in its semi-domesticated relative *Cucurbita ecuadorensis*, this trait is governed by a dominant allele *Lo-2* [35]. In zucchini, Montero-Pau et al. identified a major QTL Li_10 controlling leaf lobing [36]; Bo et al. further proposed that this trait is regulated by a pair of incompletely dominant genes and fine-mapped it to a 21 kb region on chromosome 10 [7]. However, our genetic analysis indicates that the lobed leaf trait in zucchini follows a 9:6:1 segregation ratio, suggesting that this trait is collectively controlled by two pairs of recessive alleles exhibiting epistatic effects. This finding aligns with classic genetic cases in other species, such as plant architecture in maize [37] and keratin membrane traits in semi-leafless pea [38], which similarly display the 9:6:1 ratio and digenic control. Furthermore, we identified a QTL on chromosome 10 in a genomic interval adjacent to that reported by Bo et al. [7] (Figure 2d). However, the candidate gene ultimately identified in our study, *CpARF6*, differs from their reported gene, *Cpdll* (Figure 3d, Figure 4). We speculate that such differences in the revealed genetic mechanisms may stem from the use of distinct parental varieties. Our conclusion is based on correlative expression data from the parental lines, and given the relatively limited sample size, functional validation in segregating populations remains an important next step. We also identified a second significant QTL located on the currently unassembled chromosome 0 (Figure 2d). Although the current genome assembly of zucchini hinders the precise mapping and identification of genes within this region, the discovery of this locus is a key finding that completes our initial genetic model of leaf lobation. We propose that the gene in this unassembled region contributes independently to the leaf shape and may act complementarily with the major effect gene *CpARF6* on chromosome 10 to fine-tune the final lobed phenotype. Future research will focus on constructing a telomere-to-telomere (T2T) genome and employing high-resolution genetic mapping to pinpoint the key gene governing leaf lobation within the second QTL, ultimately leading to its functional characterization.

To elucidate the function of the candidate gene *CpARF6*, we compared the genomic DNA and cDNA sequences between the two parental lines. Sequence alignment revealed variations, including 12 SNPs within the coding region, 5 of which were nonsynonymous (Figure S2). Notably, a serine-to-leucine substitution at position 335 is located within a conserved auxin response domain of the protein (Figure 5c), suggesting that this mutation may disrupt its normal function. Additionally, functional annotation confirmed that *CpARF6* encodes an ARF and exhibits homology to *AT4G30080* in *Arabidopsis*, a gene involved in leaf morphogenesis. ARFs are transcription factors that bind to AuxREs to activate or repress downstream gene expression and play a central role in compound leaf development and margin patterning [11]. In *Arabidopsis*, loss of *ARF3/ARF4* function leads to defects in leaf margin development, and the *arf2 arf3 arf4* triple mutant fails to form a proper leaf margin [12]. Similarly, in tomato, loss of *SlARF24* influences leaflet initiation by modulating *SlPIN1* expression [39]. Additionally, *SlARF10A/B* or *SlARF17* overexpression increases leaf complexity [40,41], and *SlARF3/4* are implicated in leaflet formation [42]. Furthermore, a direct regulatory relationship exists between ARF and KNOX family genes. For instance, in cotton, *GhARF16-1* directly transcriptionally regulates *GhKNOX2-1* to modulate leaf development, and genetic analysis revealed that *GhKNOX2-1* is epistatic to *GhARF16-1* even in *Arabidopsis*, indicating functional conservation [43]. This suggests that the auxin-signaling module mediating the leaf shape is likely conserved across other dicot species. Based on this cross-species evidence, we hypothesize that a similar ARF–KNOX interaction module may also operate in cucurbit crops, including zucchini. Specifically, the candidate gene *CpARF6* may regulate the expression of KNOX, thereby coordinately controlling the formation of leaf lobing in zucchini. Our future work will therefore focus on validating the biological function of *CpARF6*, paving the way for breeding zucchini varieties with enhanced yield potential and environmental resilience.

## 4. Materials and Methods

### 4.1. Plant Materials

We conducted crosses between two inbred zucchini breeding lines maintained in our laboratory: entire-leaf LR (♂) × deeply lobed Xi (♀). Self-pollination of F_1_ plants yielded an F_2_ population (392 individuals), which was subsequently selfed to generate an F_3_ population for genetic analysis. Upon maturation, the F_1_ plants were self-pollinated to generate F_2_ seeds. From these, we cultivated 392 F_2_ plants and subsequently performed self-pollination to establish the F_3_ population. All parental and progeny materials were grown at the Wuwangnong Group Research Center in Hangzhou, Zhejiang Province (coordinates: 120.659126, 30.292829) under standardized cultivation conditions featuring single-fruit retention per plant, controlled artificial pollination (for both hybridization and selfing), and uniform management of irrigation, fertilization, and pest control to maintain consistent growing environments. The lobed leaf phenotype could be visually distinguished, with entire and deeply lobed leaves exhibiting characteristic morphologies similar to the LR and Xi varieties, respectively.

### 4.2. BSA-Seq Analysis

We performed BSA analysis to identify genetic loci associated with leaf margin morphology in zucchini. From an F_2_ segregating population, we selected 30 individuals each exhibiting extreme entire-leaf or deeply lobed leaf phenotypes. Genomic DNA was extracted using the CTAB method [44] and equal amounts from each extreme group were pooled to construct L pool (lobed) and NL pool (non-lobed) bulks. Sequencing libraries were prepared with the TruSeq Nano DNA HT Kit (Illumina, San Diego, CA, USA) using 1.5 μg input DNA per sample, followed by 150 bp paired-end sequencing on an Illumina HiSeq™ PE150 platform (average insert size: 350 bp). Raw reads were quality-filtered using FASTQC [45] and aligned to the zucchini genome (http://cucurbitgenomics.org/organism/14, accessed on 1 September 2020) using BWA [46]. PCR duplicates were removed using SAMtools [47], followed by variant calling with GATK3.8 [48] and annotation using ANNOVAR (2015Dec14) [49]. The single nucleotide polymorphism index (SNP-index) was calculated for each bulk according to Takagi et al. [50], with positions showing an SNP-index <0.3 being excluded from further analysis. We performed sliding-window analysis (1 Mb window size, 10 kb step size) to identify genomic regions with significant Δ(SNP-index) differences between bulks, using 1000 permutation tests at a 95% confidence level as the significance threshold.

### 4.3. Kompetitive Allele-Specific PCR (KASP) Genotyping

KASP is a fluorescence-based high-throughput SNP genotyping technology designed for precise identification of genetic variations at the nucleotide level. The core principle of this technique lies in its allele-specific primer design. For each target SNP, two forward primers (F1 and F2) are designed 20–30 bp upstream of the SNP site, with their 3′-terminal nucleotides specifically complementary to the respective SNP alleles. These forward primers are labeled at their 5′-ends with FAM (GAAGGTGACCAAGTTCATGCT) and HEX (GAAGGTCGGAGTCAACGGATT) fluorescent tags. A common reverse primer (R) is designed 20–60 bp downstream of the SNP, ensuring an amplicon length of 80–120 bp, with the primer annealing temperatures maintained between 55–61 °C. The KASP markers used in this study are detailed in Table S1.

### 4.4. qRT-PCR Experimental Method

Total RNA was extracted from fresh plant leaves using the Easy RNA Extraction Kit (Easy-do, Hangzhou, China), and the RNA was reverse-transcribed with ReverTra Ace qPCR RT Master Mix (Toyobo, Osaka, Japan). qRT-PCR used cDNA as a template, with fluorescent staining for product detection and 2^−ΔΔCt^ analysis. cDNA stock was subjected to 2-fold gradient dilution for primer screening, and gene expression assays used appropriately concentrated cDNA. Primers (3–5 pairs per gene, with specific annealing temperatures and amplicon lengths) were designed on GenScript (https://www.genscript.com/tools/pcr-primers-designer, accessed on 20 June 2021) and screened via standard curves using zucchini reference genes [51]. With *CpActin* as the internal control and 3 biological and technical replicates each, primers were selected based on efficiency, R^2^ values, and melting curve characteristics. Differential expression was calculated using the 2^−ΔΔCt^ method [52]. The qRT-PCR primers used in this study are detailed in Table S2. For expression measurement, optimized primers and templates were used with 3 replicates and a calibration sample per plate, following the same reaction system and appropriate amplification program. IBM SPSS Statistics 27.0 was used for significance analysis and graphing.

### 4.5. Gene Cloning

The target genes were amplified by PCR using KOD OneTM PCR master Mix (Toyobo, Japan) to generate blunt-end products, which were subsequently cloned into the p-EASY Blunt zero vector (TransGen, Beijing, China). The recombinant plasmids were transformed into *Escherichia coli* competent cells via heat shock and plated on LB agar containing 100 mg/L ampicillin for overnight incubation at 37 °C. The following day, single colonies were picked and cultured in LB liquid medium with ampicillin. After the bacterial liquid became turbid, colony PCR was conducted using M13 primers and 2× Rapid Taq Master Mix (Vazyme, Nanjing, China) to screen for correct clones. Bacterial liquids with appropriately sized bands were preserved, and plasmid extraction and sequencing were performed by Hangzhou Youkang Biotechnology Co., Ltd. (Hangzhou, China).

## 5. Conlusions

In conclusion, our study demonstrates that the lobed leaf trait in zucchini is controlled by two pairs of alleles. We further identified *CpARF6* as a key candidate gene associated with this trait, potentially functioning through auxin-responsive or KNOX-mediated pathways. These findings on the lobed leaf trait provide a theoretical foundation for molecular breeding programs in zucchini. Furthermore, the genetic dissection enables the development of functional markers for selecting the ideal leaf shape, which could contribute to improved canopy structure and stress adaptation in cucurbit crops.

## Figures and Tables

**Table 1:** Separation ratio of lobed leaf traits in F_2_ population.

Offspring	Population	Entire Leaf	Shallowly Lobed Leaf	Deeply Lobed Leaf	Expected Value	χ2	χ2_0.05_
F_2_	392	205	165	22	9:6:1	3.549	5.991

Note: value χ^2^ < χ^2^_0.05_ = 5.991 represents a significant difference.

## Data Availability

All data generated in this study are included in the article and its Supplementary Information files. The BioProject ID of the BSA-seq data in NCBI is PRJNA1338379.

## Supplementary Materials

The following supporting information can be downloaded at https://www.mdpi.com/article/10.3390/ijms262010042/s1.
