# Supplementary material for: CpARF6 Controls Lobed Leaf Formation in Zucchini
Source: Int J Mol Sci. 2025 Oct 15;26(20):10042. doi: 10.3390/ijms262010042 (PMC12563625; doi:10.3390/ijms262010042)
Supplement: Supplementary file 1 [file ijms-26-10042-s001.zip › Supplementary figures.pdf]

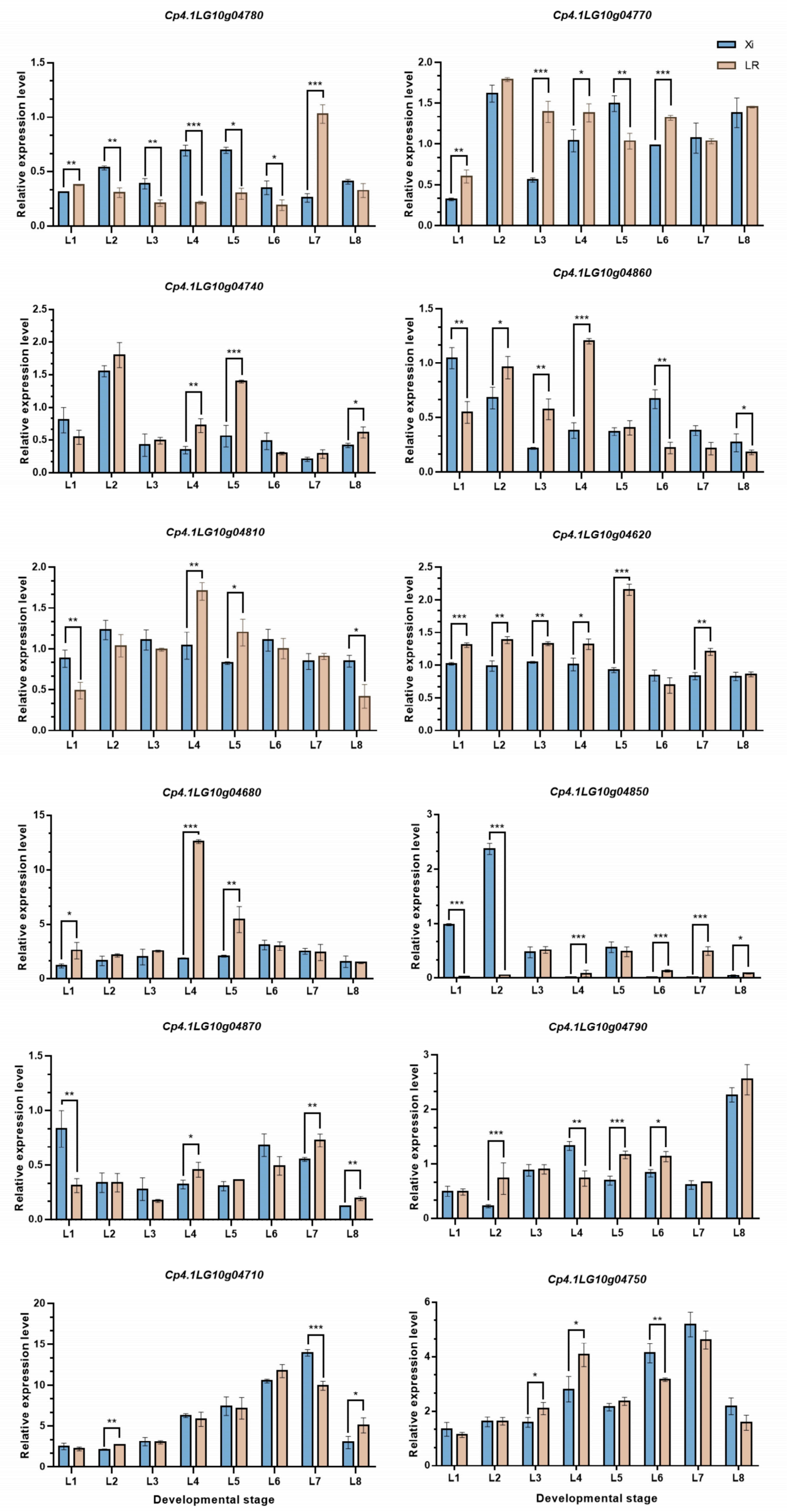

Figure S1 Gene expression levels of the candidate gene at different developmental stages.

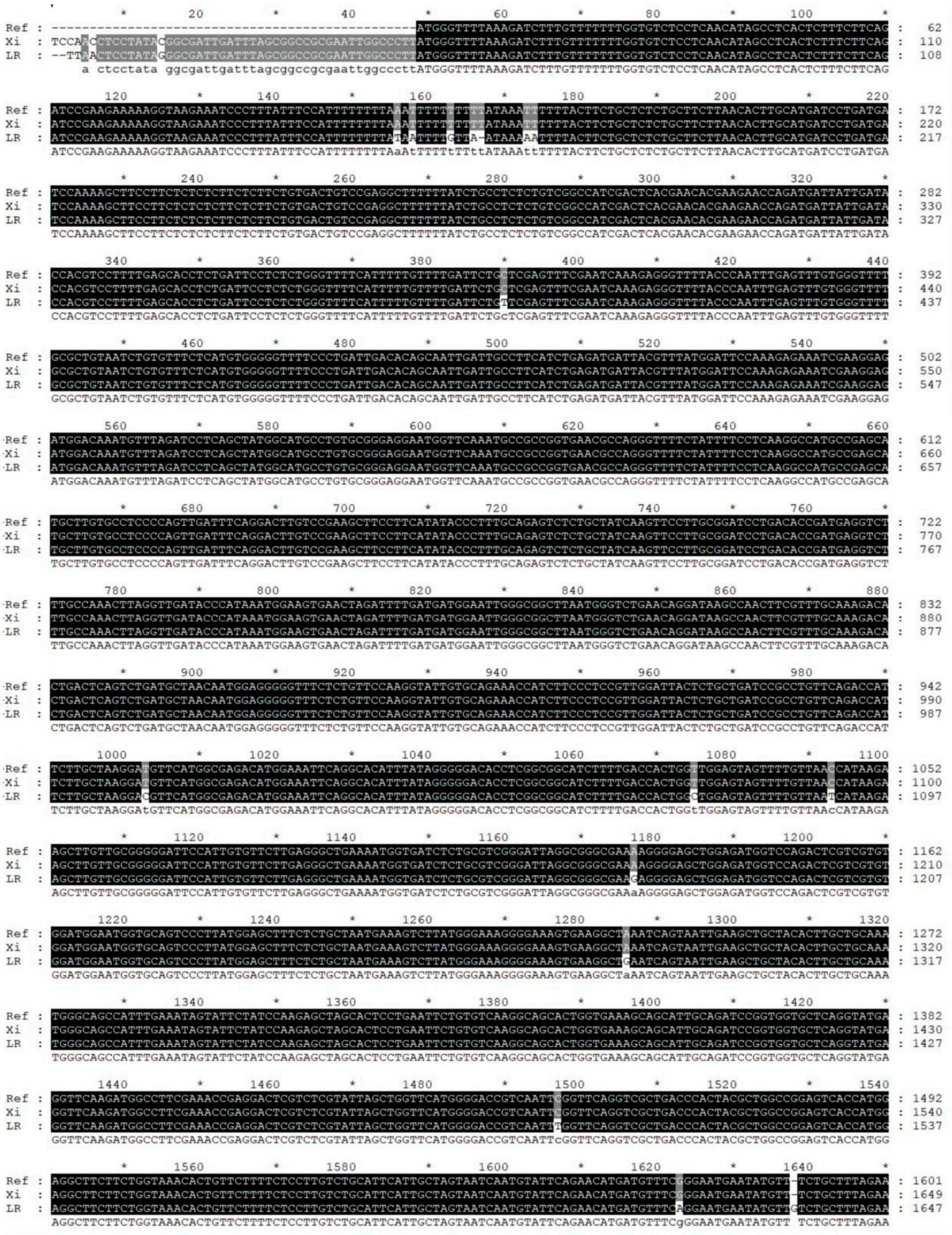

Figure S2-1 Full-length sequence alignment of the candidate gene *CpARF6* in parental lines LR and Xi

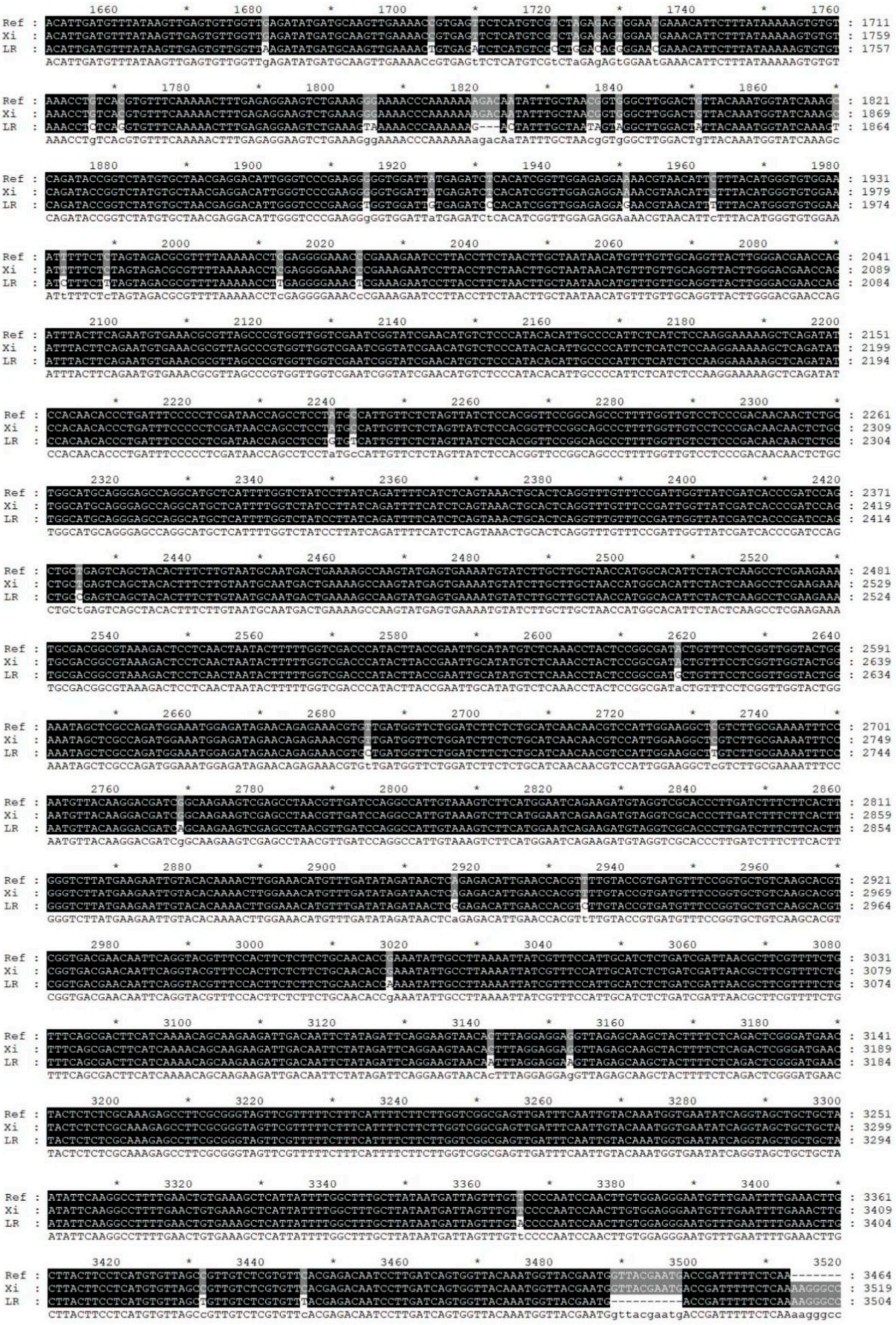

Figure S2-2 Full-length sequence alignment of the candidate gene *CpARF6* in parental lines LR and Xi

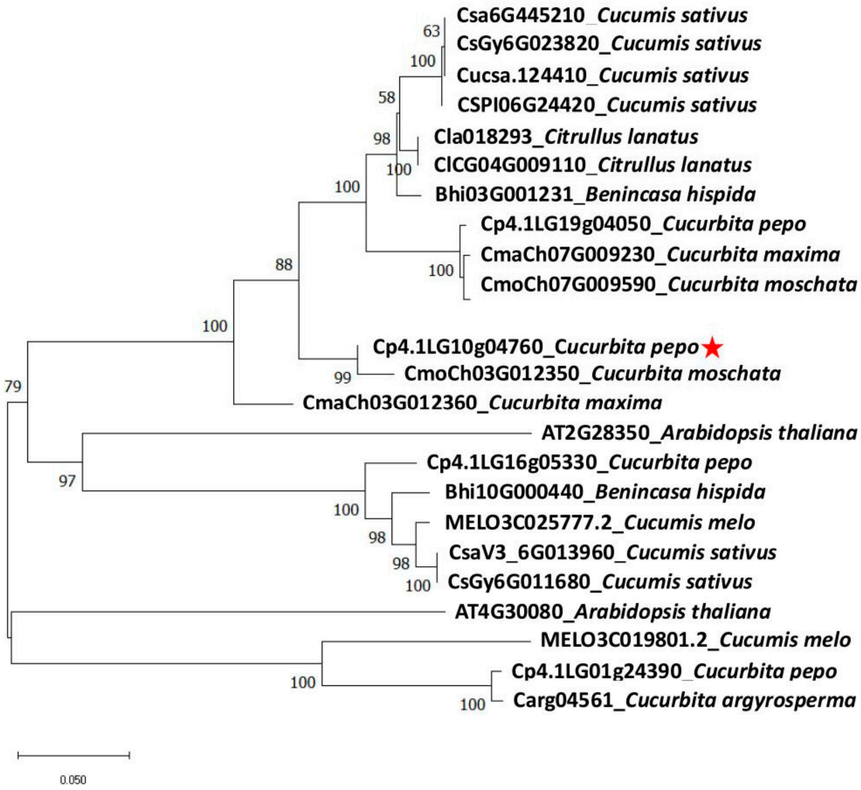

Figure S3 Phylogenetic analysis of *CpARF6* homologous gene**.**The number of nodes represents the percentage of bootstrap replication for each branch evolution, and the length of the branch line represents the degree of divergence.

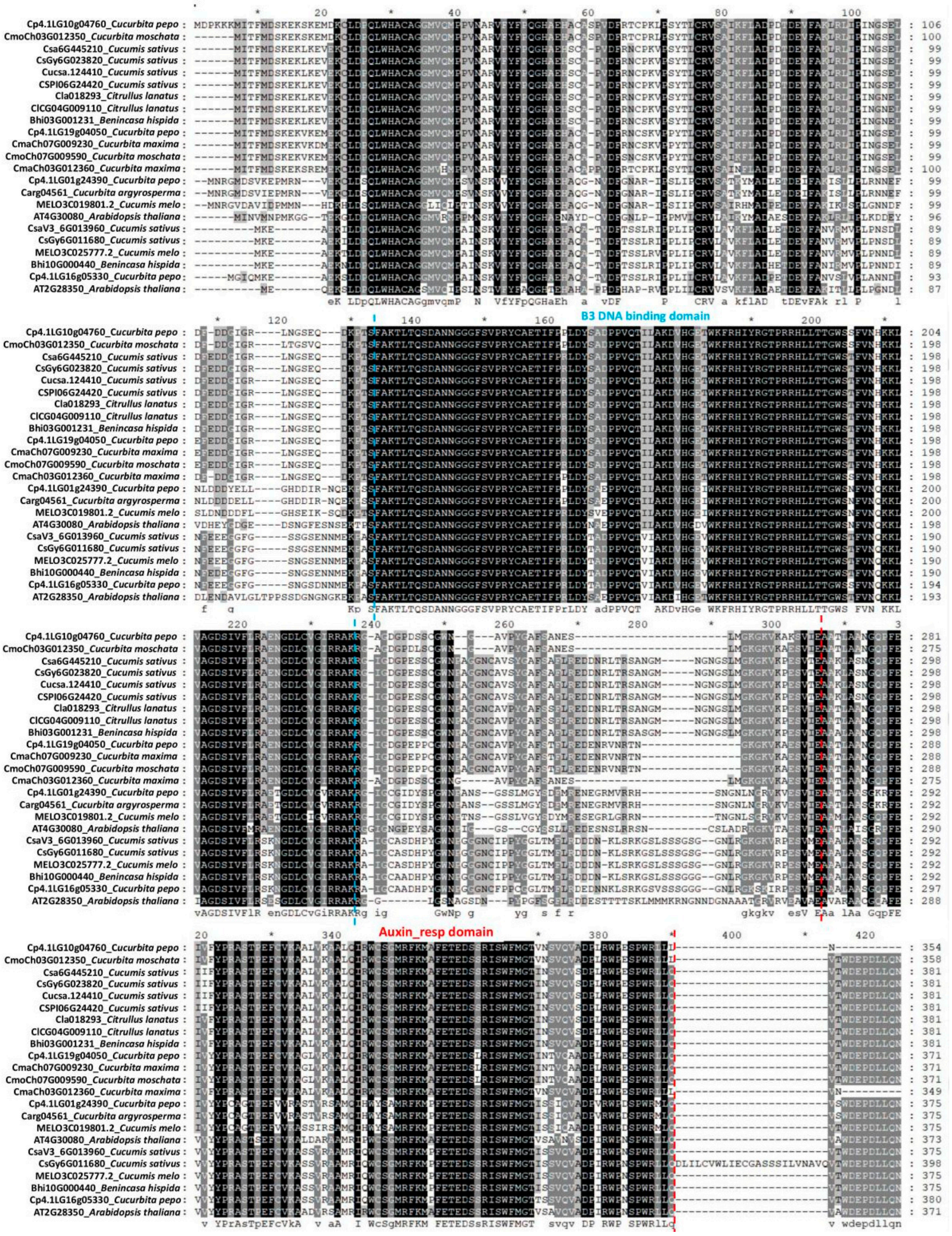

Figure S4 Alignment of amino acid sequences homologous to *CpARF6* in 8 cucurbit crops and *Arabidopsis thaliana*.
